# Consent and Internet-Enabled Human Genomics

**DOI:** 10.1371/journal.pgen.1000965

**Published:** 2010-06-24

**Authors:** Greg Gibson, Gregory P. Copenhaver

**Affiliations:** 1 Center for Integrative Genomics, School of Biology, Georgia Institute of Technology, Atlanta, Georgia, United States of America; 2 Department of Biology and the Carolina Center for Genome Sciences, University of North Carolina at Chapel Hill, Chapel Hill, North Carolina, United States of America; 3 Lineberger Comprehensive Cancer Center, University of North Carolina School of Medicine, Chapel Hill, North Carolina, United States of America

This month, *PLoS Genetics* is publishing an article from the company 23andMe reporting the first genome-wide association studies (GWAS) on multiple traits ascertained by self-reported information provided through the Internet from over 10,000 participants who pay the company for providing whole genome genotypes [1]. The paper passed through scientific review by a panel of three experts relatively quickly and is sure to attract the attention of anyone with freckles, curly hair, or an aversion to asparagus. Novel associations are described for four intrinsically interesting traits (out of 22 considered), while known associations with hair and eye color are replicated in a dynamic data-gathering context. Additionally, intriguing observations on the interaction between genetic self-knowledge and self-report of phenotypes are described. The implications of the successful application of this Internet-enabled approach to GWAS research were considered to be more than sufficient to warrant publication in the journal.

However, publication was delayed for six months while the editors sought a variety of opinions on three issues: ethical review, consent, and data access. Anyone who has read Rebecca Skloot's *The Immortal Life of Henrietta Lacks* [2] will be sensitive to the ongoing ethical and moral concerns surrounding consent and research with human samples. The editors of *PLoS Genetics* decided to proceed after satisfying ourselves on two major points, namely that the participants were not coerced to participate in the study in any way, and they were clearly aware that their samples would be used for genetic research. Recognizing that institutional review is an imperfect process and that the complexities raised by GWAS are not readily resolved, we call for a concerted effort on the part of granting agencies, scientists, review boards, and the public communities they serve to standardize processes and procedures of consent and review of human genomic research. It also needs to be stated that the Editor-in-Chief, Professor Greg Barsh, is a potential consultant to 23andMe, and so recused himself from all dealings with this paper prior to acceptance.

## Institutional Review

The first issue that attracted our attention was that the initial submission lacked a document indicating that the study had passed review by an institutional review board (IRB). The authors responded by submitting a report, obtained after the initial round of review, from the Association for the Accreditation of Human Research Protection Programs (AAHRPP)–accredited company Independent Review Consulting, Inc. (IRC: San Anselmo, CA), exempting them from review on the basis that their activity is “not human subjects research.” On the face of it, this seems preposterous, but on further review, this decision follows not uncommon practices by most scientists and institutional review boards, both academic and commercial, and is based on a guidance statement from the United States Department of Health and Human Services' Office of Human Research Protection (http://www.hhs.gov/ohrp/humansubjects/guidance/cdebiol.htm). Specifically (and as documented in part C2 of the IRC report), there are two criteria that must be met in order to determine that a study involves human subjects research: will the investigators obtain the data through intervention or interaction with the participants, and will the identity of the subject be readily ascertained by the investigator or associated with the information. For the 23andMe study, the answer to both tests was “no,” ostensibly because there was never any interpersonal contact between investigator and participant (that is, data and samples are provided without participants meeting any investigator), and the participant names are anonymous with respect to the data seen by the investigators. It follows from the logic of the IRC review, in accordance with the OHRP guidance documents, that this study does not involve human subjects research.

It is not the policy of *PLoS Genetics* to routinely delve into the specifics of individual IRB determinations, which we assume provide reasonable oversight of the process by which human samples and data are obtained. In this case, concerns were raised that a commercial IRB, paid for their opinion by the company, is not in a position of independence, but this is standard practice in the pharmaceutical and biotechnical industries, and similar concerns can be raised over the independence of University boards considering multi-million–dollar studies. Furthermore, although several of the authors have academic affiliations, we obtained express certification that the study was not performed under the auspices of their Universities, and we did not feel that review by an academic IRB was necessarily appropriate. Finally, we were also mindful of the fact that this is a minimal-risk study that would almost certainly pass institutional review contingent on the adequacy of the consent process, so next focused our attention on that aspect.

## Consent

Participants in the 23andMe study are required to sign an extensive “consent and legal agreement” document (see https://www.23andme.com/about/consent/), and they are provided with considerable information about the studies. Participation is without doubt voluntary as it is participant-initiated. The consent document states clearly that the services provided by 23andMe are not designed to diagnose disease or intended to provide medical advice and states the risks associated with obtaining unanticipated self-knowledge. A section on research indicates that samples will be used to advance the field of genetics and human health and to improve and expand future services, but also notes that prior to embarking on collaborative partnerships with other investigators and organizations additional individual consent will be sought before individual-level data is shared. However, we had a number of concerns regarding the consent, particularly pertaining to the use of technical jargon in the document that may limit understanding, ambiguity over what data participants understand will be published, and whether standard legal requirements are met by the document.

*PLoS Genetics* sought expert opinion and engaged in discussions among the editors. We found broad agreement that if formal review of the document had been carried out under the auspices of an IRB, changes are likely to have led to an improved consent process. Some serious objections were raised, and these varied across reviewers. A major one related to the definition of collaboration and the listing of one of the senior authors solely as an affiliate of Columbia University, implying that he was a collaborator and hence that independent consent may have been required. However, the authors have confirmed that his participation was as a consultant to 23andMe, with a core role in study planning and analysis. On balance, the editors of *PLoS Genetics* were satisfied that the document meets minimal legal requirements and that there is sufficient information for participants to realize that they are participating in genetic research (if not human subjects research), that there are associated risks, and that study conclusions will be published with every effort made to protect participant anonymity and restrict access to their own genotypes.

We then had to decide whether to require re-consent of over 3,000 participants. Practically, this would be a formidable task, although, given the Web-based nature of the study, automated contact with the vast majority of participants would be possible. Furthermore, a formal rewriting of the document that satisfied all possible concerns, given the diversity of opinion we encountered, would take considerable time. Consequently, we elected to require that the authors address our concerns about their consent process moving forward, and they now indicate in the published paper that IRC has since been fully engaged as a formal IRB. We also note that the experience of 23andMe reflects an unfortunate loophole that applies to all research with human samples that is not, as above, formally designated to be “human subjects research.” For situations in which a study does not meet the aforementioned criteria but obtaining a consent form would still be desirable, there are no guidelines or policy with regard to how such a consent form should be developed and reviewed in an ethically responsible manner.

## Data Access

Our third major issue concerned data access. The desire to promote open access to data is complicated by the evolving difficulty in protecting the identity of study participants who provide whole genome data. It is now apparent that someone with access to an individual's whole genome genotype data can, in theory, determine whether they were a member of a group given just the aggregate (that is, summary allele frequency) data for that group [3], [4]. The authors of the study now reported [1] have provided limited aggregate data related to their statistically significant genetic associations with traits, as they implied they would in their consent document, and we note that these data are insufficient to identify participants.

Current policy of the NIH, Wellcome Trust, and other large-scale public human genotyping efforts is to restrict access to individual genotypes to permitted expert investigators, while encouraging submission of the individual profiles to public repositories. Such submission is precluded by the consent obtained by 23andMe, and we agree that it would be unreasonable to require it. Individuals who voluntarily participate in commercial-sponsored research should not be asked to agree to have their personal genomic data submitted to a government-sponsored data repository, no matter what access restrictions are currently in place. Having paid for the service, they have a reasonable expectation that their personal information will not be provided to the general public without specific consent. This places *PLoS Genetics* in the position of promoting open access to the research enterprise, but having to decide on the appropriateness of publishing a study where access is more restricted than usual. In this decision, we must balance the public good of open access to research with the public good of disseminating valuable science performed by commercial entities. Noting that there is potential for access to the underlying data for collaborators through a re-consent process, on balance we decided that the interests of presenting the findings to the genetics community favored publication of the study.

## Summary Statement

The editors of *PLoS Genetics* recognize that the decision to publish this study, without IRB review as human subjects research and with some concerns over the consent document, and the fact that there is limited access to the raw data, will not sit well with some, perhaps many, readers. As outlined above, though, a *prima facie* valid IRB exemption was obtained, and, while there are ambiguities in the consent form, there was no evidence that these amount to an inadequate document. After considering all of the evidence, we decided that publication, accompanied by an editorial providing transparent documentation of the process of consideration, was the most appropriate course.

In so doing, we call for community input to spur efforts to standardize the IRB consent process for GWAS research. With a few exceptions, academic IRBs are not typically constituted by geneticists and certainly not by experts with expertise in contemporary genomic profiling and all of the issues it raises. Current practice follows norms established in an era when studies involved dozens, or maybe hundreds, of participants and focused on one or a few biomarkers. We now face the prospect in the coming decade of whole-genome sequence data obtained for thousands of individuals on standard individual–investigator research grants. It is almost inconceivable that even scientifically literate members of the public will appreciate the full implications of the provision of whole-genome genetic data, yet we must trust participants to make informed and sensible decisions. At the same time, consent documents necessarily simplify very difficult genetic issues that even experts disagree over and use lay language that glosses over technical matters. A good argument can be made that the consent process followed by 23andMe study participants, presumably following considerable reflection, is more informed than most processes that have been formally reviewed. Against this background, we have had extensive discussion with the authors of this study to address our concerns and to update their processes, but we anticipate broad evolution of GWAS consent and review in the near future.
